# Survival After Simultaneous Pancreas‐Kidney Transplantation in Type 1 Diabetes: The Critical Role of Early Pancreas Allograft Function

**DOI:** 10.3389/ti.2022.10618

**Published:** 2022-09-12

**Authors:** Mengmeng Ji, Mei Wang, Wenjun Hu, Mohamed Ibrahim, Krista L. Lentine, Massini Merzkani, Haris Murad, Yazen Al-Hosni, Ronald Parsons, Jason Wellen, Su-Hsin Chang, Tarek Alhamad

**Affiliations:** ^1^ School of Medicine, Washington University in St. Louis, St. Louis, MO, United States; ^2^ Division of Nephrology, Department of Internal Medicine, Saint Louis University School of Medicine, St. Louis, MO, United States; ^3^ Division of Transplantation, Department of Surgery, Emory University School of Medicine, Atlanta, GA, United States

**Keywords:** allograft failure, kidney transplant, simultaneous pancreas-kidney transplantation, type 1 diabetes mellitus, allograft survival

## Abstract

Simultaneous pancreas-kidney transplantation (SPK) carries about a 7%–22% risk of technical failure, but the impact of early pancreas allograft loss on subsequent kidney graft and patient survival is not well-defined. We examined national transplant registry data for type 1 diabetic patients who received SPK between 2000 and 2021. Associations of transplant type (i.e., SPK, deceased‐donor kidney transplant [DDKA], living‐donor kidney transplant [LDKA]) with kidney graft failure and patient survival were estimated by multivariable inverse probability of treatment-weighted accelerated failure-time models. Compared to SPK recipients with a functioning pancreas graft 3 months posttransplant (SPK,P+), LDKA had 18% (Time Ratio [TR] 0.82, 95%CI: 0.70–0.95) less graft survival time and 18% (TR 0.82, 95%CI: 0.68–0.97) less patient survival time, DDKA had 23% (TR 0.77, 95%CI: 0.68–0.87) less graft survival time and 29% (TR 0.71, 95%CI: 0.62–0.81) less patient survival time, and SPK with early pancreas graft loss had 34% (TR 0.66, 95%CI: 0.56–0.78) less graft survival time and 34% (TR 0.66, 95%CI: 0.55–0.79) less patient survival time. In conclusion, SPK,P+ recipients have better kidney allograft and patient survival compared with LDKA and DDKA. Early pancreas graft failure results in inferior kidney and patient survival time compared to kidney transplant alone.

## Graphical Abstract

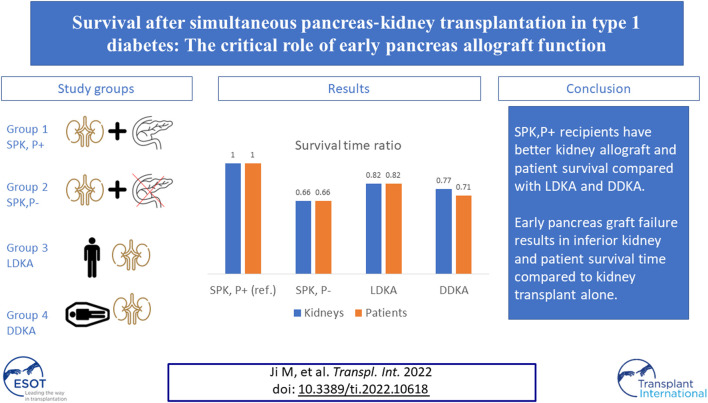

## Introduction

In the United States, 60.6% of incident patients with end-stage kidney disease (ESKD) have diabetes mellitus (DM) (1). Mortality rates of patients with ESKD and DM vary depending on treatment choice. Those on dialysis have a 15%–20% mortality rate within 1 year of treatment initiation and a 5-year survival rate of under 50% (1). Compared to diabetic patients on dialysis, those who receive kidney transplants have significantly higher 5-year survival rates of 85% for type 1 diabetes (T1DM) and 77% for type 2 diabetes (T2DM) (2). However, poor glycemic control after transplantation remains an important challenge and contributes to excessive morbidity and mortality among diabetic recipients (3–5). Simultaneous pancreas-kidney (SPK) transplantation is a well-established treatment for patients with T1DM to restore normoglycemia and ameliorate diabetic complications (6). Owing to improved surgical technique, immunosuppression, donor and recipient selection, and graft surveillance, the 5- and 10-year patient survival rates for SPK transplantation have reached 87% and 70%, respectively (7).

Previous studies reported conflicting results about whether long-term kidney allograft and patient survival in SPK recipients is superior to that of kidney transplant alone recipients, especially as compared with a living donor kidney transplant alone (LDKA) (8–10). The mixed results may partially attribute to the higher rate of postoperative complications associated with SPK and the long‐term benefits of euglycemia afforded by a functioning pancreas allograft. Despite improvements in surgical technique, the recent Scientific Registry of Transplant Recipients (SRTR) report showed that roughly 7% of pancreas grafts after SPK transplant are lost within 3 months of transplant (11). Early pancreas graft loss was historically associated with reduced kidney allograft function and inferior survival outcomes (12–16). Given improvements in immunosuppression management of complications and comorbidities, studies of contemporary cohorts have reported better outcomes and excellent kidney allograft function following early pancreas loss compared to earlier studies (17). The question remains whether SPK with and without early pancreas graft function has better survival in comparison to kidney transplant alone in the current transplant era.

This study aims to determine whether conditional 3-month pancreas graft survival is associated with long‐term kidney allograft survival and patient survival in patients with T1DM who received SPK, compared to deceased donor kidney transplant alone (DDKA) and LDKA recipients, in a large, contemporary national U.S. cohort.

## Patients and Methods

### Study Population

Data was obtained from the Organ Procurement and Transplantation Network (OPTN), a data system that contains all national data on the candidate waiting list, organ donation and matching, and transplantation. We performed a retrospective cohort study of all adult ESKD patients with T1DM who received transplants (i.e., deceased donor kidney transplant [DDKA], LDKA, and SPK) between January 1, 2000 and May 31, 2021. ESRD due to T1DM was defined based on the diagnosis as reported by transplant centers to UNOS, where the diabetic status of recipients was categorized into six groups: no diabetes, type 1, type 2, other type, type unknown, and missing. The proportion of unknown type and missing data is small (<1%). Exclusion criteria included: 1) younger than 18 years of age at the time of transplant, 2) multiorgan transplants aside from SPK, and 3) previous KT recipients. In addition, patients who died or developed kidney allograft failure within 3 months of transplant were excluded. Surviving SPK recipients with kidney allograft function at 3 months were further categorized into two groups: (1) SPK with a functioning pancreas graft at 3 months posttransplant (SPK, P+); and (2) SPK recipients with a loss of pancreas graft at 3 months posttransplant (SPK, P‐). We additionally evaluated the study population without excluding patients who died or developed kidney allograft failure within 3 months of transplant in sensitivity analyses.

### Outcomes

The outcomes were kidney allograft failure and patient death. For kidney allograft failure, the survival time was calculated from the date of transplantation to the date of irreversible graft failure signified by a return to dialysis, kidney re-transplantation, or patient death. For patient survival, patients were followed until death or being censored. Patient outcomes were followed‐up until September 2021.

### Statistical Analysis

Descriptive data were summarized as percentage (%) for categorical variables, and differences across transplant groups were compared using the chi-squared test. Accelerated failure time (AFT) models were performed in this study, because Scaled Schoenfeld Residuals indicated a violation of proportional hazards assumption in the Cox proportional hazards models (18). The Weibull distribution was selected for AFT models based on the minimum Akaike Information Criterion (AIC) among different survival distributions (i.e., exponential, loglogistic, Weibull, and lognormal). A multivariable AFT model was adjusted for recipient factor (age, gender, race, body mass index [BMI], dialysis time, panel reactive antibody [PRA], donor/recipient cytomegalic virus [CMV] serostatus), donor (age, gender, race, BMI, hypertension status), and transplant factors (cold ischemia time, human leukocyte antigen [HLA] mismatch). The results of AFT models were expressed in acceleration coefficients, which explain how much faster or slower the event of interest occurred in each group. For interpretability, the results of AFT models are exponentiated to calculate time ratio (TR), which was interpreted as the expected time to graft failure or patient death in one category relative to the referent group. Unlike the interpretation of proportional hazard model results where hazard ratios larger than 1 are equal to higher risk, TRs larger than 1 were considered to have a longer survival time compared to the reference group.

To account for the potential bias arising from confounding variables that affect the selection of patients into different groups and the outcomes, generalized boosted regression with covariates (see Table 1) was performed to predict a patient’s propensity score of receiving a certain type of transplant, which was then used to generate the weights for the inverse probability of treatment weighted (IPTW) Kaplan-Meier curves and IPTW AFT models. Covariate balance was assessed by comparing the absolute standardized mean differences (ASMD) between the treatment groups on the pretreatment covariates before and after weighting (see Supplementary Figure S1). In addition, Bonferroni correction was applied to adjust for multiple comparisons.

**TABLE 1:** Baseline recipient, donor, and transplant factors of the study cohort, stratified by transplant type.

	SPK	SPK,P+	SPK,P-	DDKA	LDKA	*p*-value
	*n* = 10,383	*n* = 9,832	*n* = 551	*n* = 6,202	*n* = 5,673	
	46.65%	44.17%	2.48%	27.86%	25.49%	
Recipient factors						
Age (years)^a^ ^,^ ^b^						<0.0001
18–50	84.11	84.07	84.75	47.55	62.08	
>50	15.89	15.93	15.25	52.45	37.92	
Gender^a^ ^,^ ^b^						<0.0001
Female	39.20	39.10	41.02	42.62	42.73	
Male	60.80	60.90	58.98	57.38	57.27	
Race^a^ ^,^ ^b^						<0.0001
White	65.70	65.57	68.06	57.59	78.48	
Black	19.91	19.97	18.87	24.22	8.81	
Hispanic	11.62	11.70	10.16	13.74	10.31	
Other	2.77	2.77	2.90	4.45	2.40	
BMI (kg/m^2^)^a^ ^,^ ^b^ ^,^ ^c^						<0.0001
<18.5	1.96	1.97	1.81	1.47	1.89	
18.5–24.9	50.11	50.47	43.74	30.81	40.67	
25–29.9	36.25	36.26	36.12	32.65	32.15	
>30	11.53	11.16	18.15	34.76	24.55	
PRA%^a^ ^,^ ^b^						<0.0001
0	70.29	70.36	68.97	54.11	58.12	
1–19	12.87	12.89	12.52	13.03	11.9	
20–80	12.62	12.56	13.61	16.37	10.59	
>80	3.40	3.36	4.17	15.41	3.21	
Missing	0.83	0.83	0.73	1.08	16.18	
Dialysis time^a^ ^,^ ^b^						<0.0001
0	20.47	20.38	21.96	12.24	33.83	
<24	37.73	25.52	25.59	34.41	12.06	
24–60	25.52	37.93	34.3	17.28	30.79	
>60	5.63	5.6	6.17	25.19	2.12	
Missing	10.64	10.57	11.98	10.88	21.21	
CMV^a^ ^,^ ^b^						<0.0001
D + R+	18.67	18.66	18.87	13.87	0	
D-R-	12.67	12.6	13.97	9.88	0	
R+	29.79	29.58	33.58	41.23	36.59	
Missing	38.86	39.16	33.58	35.02	63.41	
Donor factors						
Age (years)^a^ ^,^ ^b^ ^,^ ^c^						<0.0001
<18	19.65	19.98	13.79	9.06	0	
18–50	79.64	79.38	84.21	65.64	73.17	
>50	0.71	0.64	2.00	25.30	26.83	
Gender^a^ ^,^ ^b^						
Female	30.14	30.19	29.22	39.62	62.10	
Male	69.86	69.81	90.78	60.38	37.90	
Race^a^ ^,^ ^b^						<0.0001
White	63.14	63.12	63.52	71.56	79.76	
Black	18.72	18.70	19.06	12.46	7.65	
Hispanic	14.32	14.31	14.52	12.98	10.17	
Other	3.81	3.86	2.90	3.00	3.00	
BMI (kg/m^2^)^a^ ^,^ ^b^ ^,^ ^c^						<0.0001
<18.5	6.45	6.43	7.99	6.9	0.93	
18.5–24.9	56.93	57.30	50.27	34.15	32.22	
25–29.9	29.91	29.65	34.66	30.89	40.86	
>30	6.62	6.54	7.99	29.76	22.60	
Hypertension^a^ ^,^ ^b^ ^,^ ^c^						<0.0001
No	95.58	95.72	93.1	73.49	97.17	
Yes	4.42	4.28	6.9	26.51	2.83	
Transplant factor						
HLA Mismatch^a^ ^,^ ^b^						<0.0001
0	0.72	0.71	0.91	12.16	8.5	
1–2	3.57	3.52	4.54	7.79	18.58	
3–6	95.70	95.77	94.56	79.49	71.81	
Cold ischemic time (hours)^a^ ^,^ ^b^ ^,^ ^c^						<0.0001
<12	60.59	60.91	54.81	24.83	80.2	
12–24	33.81	33.51	39.2	53.92	1.23	
>24	1.32	1.32	1.27	17.98	0.76	
Missing	4.28	4.25	4.72	3.27	17.8	

a *p* < 0.05 for chi‐squared tests comparing differences between SPK,P+, SPK,P‐, DDKA, and LDKA groups.

b *p* < 0.05 for chi‐squared tests comparing differences between SPK, DDKA, and LDKA groups.

c *p* < 0.05 for chi‐squared tests comparing differences between SPK,P+ and SPK,P‐ groups.

*p*-value was reported for testing differences between SPK, DDKA, and LDKA groups.

BMI, body mass index; CMV, cytomegalovirus; DDKA, deceased‐donor kidney transplant alone; HLA, human leukocyte antigen; LDKA, living‐donor kidney transplant alone; PRA, panel reactive antibody; SPK, simultaneous pancreas‐kidney transplantation; SPK,P+, simultaneous pancreas‐kidney transplant recipients with a functioning pancreas graft at 3‐month post‐SPK; SPK,P‐, simultaneous pancreas‐kidney transplant recipients with loss of pancreas graft at 3‐month post‐SPK.

All statistical analyses were performed using STATA 15.1 version (StataCorp, College Station, TX) and TWANG package in R statistical software version 4.0.2 (R Project for Statistical Computing).

### Ethical Statements

Exemptions for study approval and informed consent were obtained for this cohort study from the Washington University in St. Louis School of Medicine Institutional Review Board because the study was secondary analyses of deidentified data.

### Results

**FIGURE 1:**
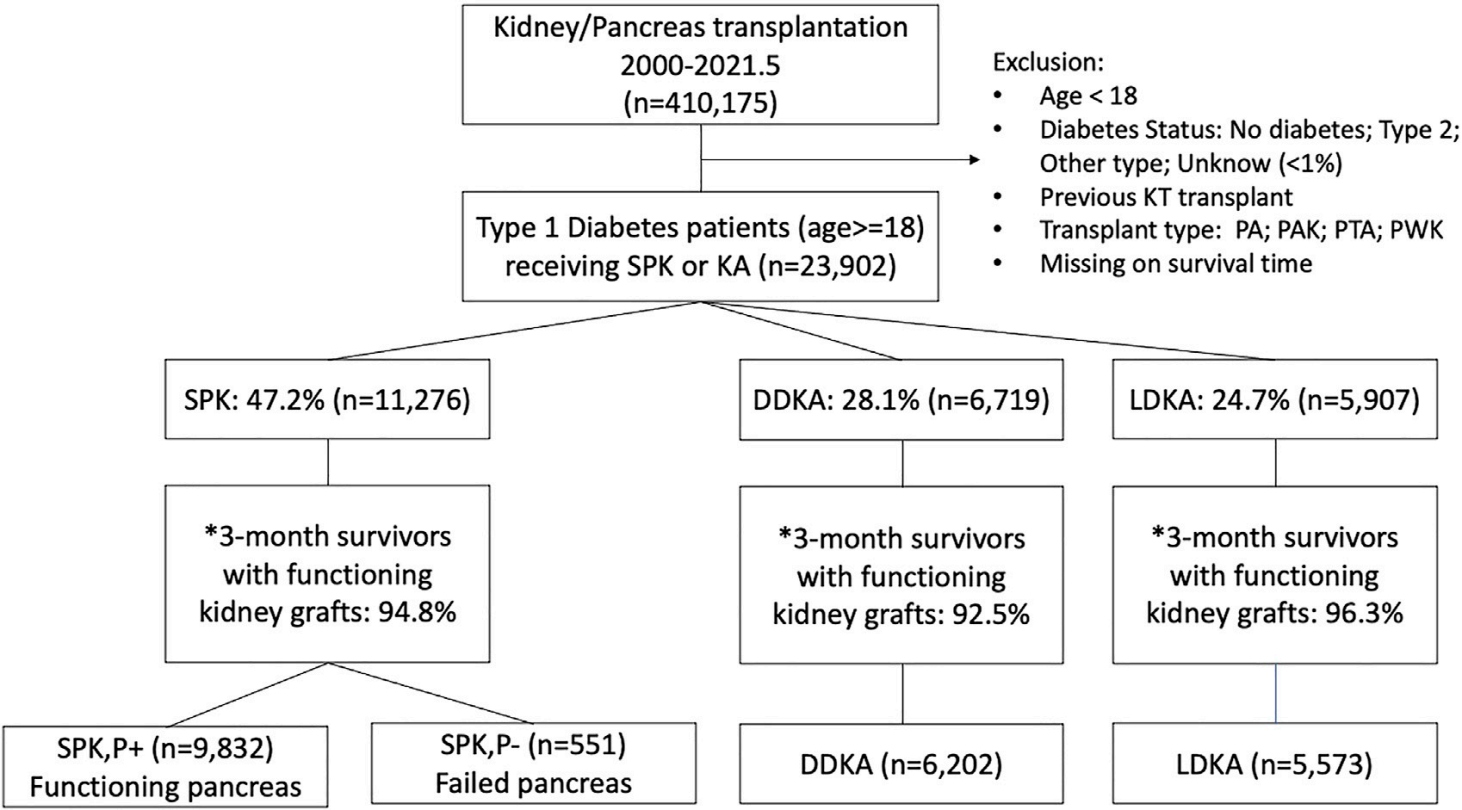
Sampling scheme for identification of kidney transplants in recipients with kidney disease secondary to type 1 diabetes from 2000 to 2021.

Figure 1 shows the sampling scheme for identification of adult (age ≥18 years) patients with T1DM who received SPK (*n* = 11,276), DDKA (*n* = 6,719), or LDKA (*n* = 5,907) between 2000 and 2021. Among them, 94.8% of the SPK patients (*n* = 10,383), 92.5% of the DDKA patients (*n* = 6,202), and 96.3% of the LDKA patients (*n* = 5,673) survived with functioning kidney grafts within 3 months following transplantation. Early pancreas loss within 3 months occurred in 6.4% of SPK recipients. Among these 10,383 SPK recipients with functioning kidney at 3 months, 5.3% had pancreas allograft failure within 3 months (SPK,P‐, *n* = 551), and the remaining 94.7% had a functioning pancreatic graft at 3 months (SPK,P+, *n* = 9,832).

Table 1 shows the baseline recipient, donor, and transplant factors of 22,258 transplants stratified by transplant type. These were statistically significantly different across transplant types. Notably, SPK recipients were more likely to be younger (age ≤50), male, and with a normal weight, but less likely to have zero HLA mismatches than kidney transplant alone recipients. DDKA recipients had higher PRA (PRA>80) and longer dialysis time (>60 months) compared to SPK and LDKA recipients. Donors for SPK were more likely to be younger (age ≤50), male, within the normal weight, and have shorter cold ischemia times (<24 h). Donors for DDKA were more likely to be hypertensive. Compared with SPK,P- recipients, SPK,P+ recipients were more likely to have normal BMI. Donors of SPK,P- recipients were more likely to be older, obese, hypertensive, and had longer cold ischemia times than donors of SPK,P+.

**FIGURE 2:**
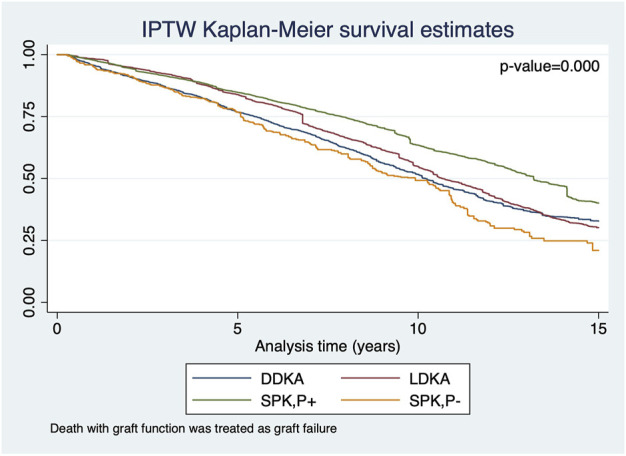
IPTW Kaplan-Meier curves for kidney allograft survival in recipients who survived the first 3 months of transplant with functioning kidney allograft.

**FIGURE 3:**
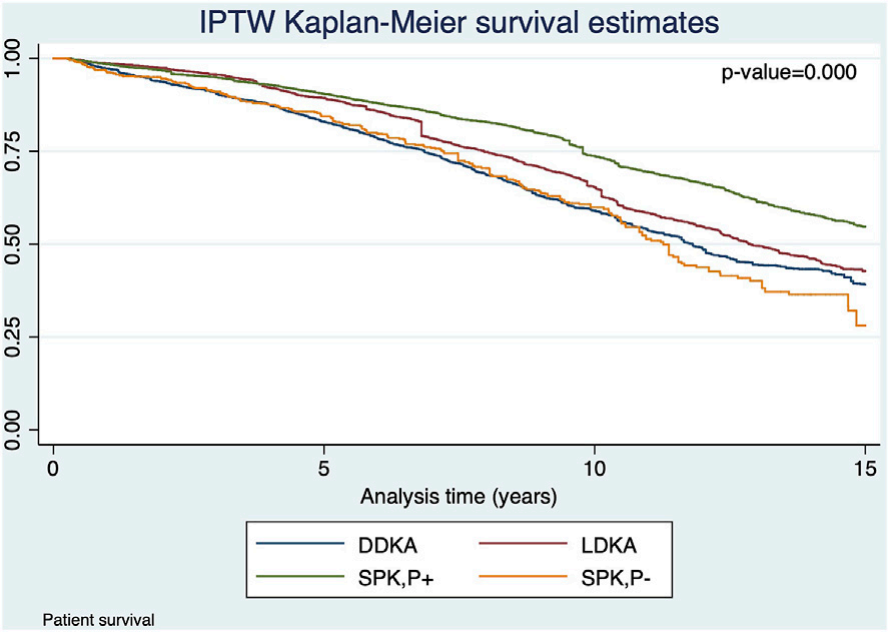
IPTW Kaplan-Meier curves for patient overall survival in recipients who survived the first 3 months of transplant with functioning kidney allograft.

The Kaplan‐Meier curves of SPK,P+ and LDKA recipients crossed during early years post-transplant. In the long-term, SPK,P+ recipients showed better kidney allograft survival and patient survival than LDKA, SPK,P- and DDKA recipients (Figures 2, 3). Table 2 presented results from multivariable-adjusted AFT models. Compared to SPK,P+ recipients, LDKA had 18% less graft survival time (TR 0.82, 95% Confidence Interval [CI]: 0.70, 0.95) and 18% less patient survival time (TR 0.82, 95% CI: 0.68, 0.97), DDKA had 23% less graft survival time (TR 0.77, 95% CI: 0.68, 0.87) and 29% less patient survival time (TR 0.71, 95% CI: 0.62, 0.81), and SPK,P- had 34% less graft survival time (TR 0.66, 95% CI: 0.56, 0.78) and 34% less patient survival time (TR 0.66, 95% CI: 0.55, 0.79). When including patients who died or developed kidney allograft failure within 3 months of transplant, the results for kidney and patient survival were similar.

**TABLE 2:** Model results of multivariable inverse probability of treatment-weighted (IPTW) weibull accelerated failure time (AFT) for graft failure and patient death.

Event	Time ratio [95% bonferroni-adjusted CI]	
	Kidney graft failure	Patient death
	Main analyses (References group: SPK,P+)	
LDKA	0.82 [0.70, 0.95]**	0.82 [0.68, 0.97]*
DDKA	0.77 [0.68, 0.87]***	0.71 [0.62, 0.81]***
SPK,P-	0.66 [0.56, 0.78]***	0.66 [0.55, 0.79]***
	Sensitivity analyses: without excluding patients who died or developed kidney allograft failure within 3 months of transplant (References group: SPK,P+)	
LDKA	0.75 [0.59, 0.95]***	0.72 [0.58, 0.90]**
DDKA	0.64 [0.51, 0.79]***	0.62 [0.51, 0.76]***
SPK,P-	0.35 [0.25, 0.49]***	0.36 [0.27, 0.49]***

Note: The results of AFT models are exponentiated to calculate time ratios, which was interpreted as the expected time to graft failure or patient death in one category relative to the referent group. Multivariate analysis was adjusted for recipients’ factors (age, gender, race, BMI, dialysis time, panel reactive antibody, donor/recipient cytomegalic virus serostatus), donors’ factors (age, gender, race, BMI, hypertension status), and transplant factors (cold ischemia time, human leukocyte antigen mismatch). *p < 0.05; **p < 0.01; ***p < 0.001.

## Discussion

This study analyzed the characteristics and outcomes of patients with kidney failure from T1DM who received kidney transplants from 2000 to 2021 in the United States. Overall, 47% received SPK transplants, 25% received LDKA, and 28% received DDKA. Early pancreas loss occurred in 6.4% of SPK recipients. After adjusting for propensities of the type of transplant and recipient, donor, and transplant factors captured in the transplant registry, we observed superior outcomes for kidney graft and patient survival with SPK,P+ over LDKA and DDKA. SPK recipients with early pancreas graft failure was associated with inferior kidney and patient survival compared to kidney transplant alone.

Our study found that Kaplan-Meier survival curves crossed over in the early posttransplant years and the proportional hazard assumption was violated. The survival curves showed that LDKA was associated with the best initial graft and patient survival, but long-term follow-up beyond 5 years after transplantation showed highest kidney graft and patient survival among the SPK,P+ group. This time-dependent difference in the relative survival advantage of SPK and LDKA was also found in previous studies of large transplant registries. A prior 72-month follow-up study found that LDKA was associated with significantly lower risks of kidney graft failure and patient death, while another study using the same database with longer follow-up demonstrated equivalent patient survival in SPK and LDKA recipients (9, 16). The initially better graft survival in LDKA compared with SPK may be attributed to a lower rate of delayed graft function, better HLA matching, a shorter dialysis time, a lower rate of technical problems, and lower early mortality. This result added to the evidence that differences in outcome between SPK and kidney transplantation alone can be evaluated in a valid manner only with >5 years of post-transplantation follow-up (8, 19).

Our findings of superior outcomes of SPK,P+ are similar to prior studies (12, 20). Weiss et al. found best patient survival in SPK recipients with functioning pancreas graft at 12 months posttransplant in the 1997–2005 US cohort (12). Barlow et al. found best patient survival in SPK recipients with a functioning pancreas graft at 3 months posttransplant in the 2001–2014 UK cohort (20). The survival advantage of SPK,P+ is potentially due to the long‐term euglycemic effects of a functional pancreas graft. In addition, studies have found that recipients of SPK with a functioning pancreas have a lower risk of long-term cardiovascular mortality, which is the leading cause of death in kidney transplant recipients (21, 22). Furthermore, in this study, SPK recipients were on average younger, leaner, and less likely to have hypertension. These favorable recipient and donor factors may reflect an inherent bias by transplant centers to list candidates for SPK with lower preoperative risks. Noteworthy, we performed IPTW survival analyses to control for the nonrandom assignment to different transplant types and the aforementioned factors, such as age and BMI, have been accounted for, which advances the existing studies. Nonetheless, the unobserved confounding may impact this assignment and thus overestimate the benefit of SPK.

Another main finding of this study was that SPK recipients with early technical failure of the pancreas have significantly inferior kidney graft and patient survival outcomes compared to LDKA and DDKA, although the negative influence was mitigated in subgroup recipients with >5 years of post-transplantation follow-up. The difference can be explained in large part because of complications associated or resulting from loss of the pancreas graft, including hemorrhage, sepsis, pancreatitis, thrombosis, and systemic inflammatory response (23). In SPK recipients, avoiding early technical failure of the pancreas is of great importance to avoid associated kidney graft loss, which may be achieved through improved surgical technique, proper immunosuppression, appropriate donor and recipient selection, and early detection of graft failure.

Most previous studies have concluded that living kidney donation shows better graft survival than those from deceased donors (24, 25). Surprisingly, this study did not find a significant difference in kidney graft survival between LDKA and DDKA. There are several possible explanations. First, the frequency of acute rejection episodes is lower among LDKA, which reduces chronic rejection and thereby increases long-term graft survival (26). By excluding those patients who died or developed kidney allograft failure within 3 months posttransplant, this study likely underestimated the long-term survival benefit of LKDA. Second, this analysis has controlled for recipient age, pretransplant diabetes mellitus, pretransplant PRA, donor race, sex, hypertension, and preservation-related factors such as long cold ischemia, all of which are major risk factors of graft survival rates for LDKA and DDKA (27). Additionally, we performed IPTW survival analyses to control for the nonrandom assignment to different transplant types and thus the potential bias caused by healthier LDKA recipients and donors is reduced.

Several limitations of our study should be noted. First, this was a retrospective study that can identify associations but not prove causation; therefore, the results should be interpreted carefully. Second, there was a small amount of missing data for certain factors, although we attempted to reduce their impact by adjusting for “missing” status. Third, selection bias may have occurred at the time of listing, especially as candidates with higher surgical risks may have been more suitable for kidney transplant. Although generalized boosted regression was used to estimate propensity scores to improve covariate balance across the groups, sufficient overlap of the scores across groups is not guaranteed. Last, despite the long follow‐up of this study, this database did not allow for the tracking of diabetic complications, such as retinopathy and neuropathy, and their consequential impact on quality of life. These complications may be curtailed by a functioning pancreas graft as long-term benefits in addition to graft and patient survival. Future studies addressing these issues are warranted.

In conclusion, SPK recipients with early functioning pancreas had superior kidney allograft and patient survival compared with SPK,P-, LDKA, and DDKA recipients. Our findings highlight the long‐term benefits of SPK utilization; however, the benefits are dependent on early pancreas allograft function. More research is needed to identify surgical and medical factors that increase the risks for early pancreas graft loss, such as high pancreas donor risk index and longer preservation time, to optimize patient outcomes.

## Data Availability

Publicly available datasets were analyzed in this study. This data can be found here: STAR (Standard Transplant Analysis and Research) files. https://optn.transplant.hrsa.gov/data/view-data-reports/request-data/data-request-instructions/.

## Supplementary Material

Supplementary Figure S1 Balance before and after weighting.
