# Corrigendum: Survival After Simultaneous Pancreas-Kidney Transplantation in Type 1 Diabetes: The Critical Role of Early Pancreas Allograft Function

**DOI:** 10.3389/ti.2022.10952

**Published:** 2022-11-07

**Authors:** Mengmeng Ji, Mei Wang, Wenjun Hu, Mohamed Ibrahim, Krista L. Lentine, Massini Merzkani, Haris Murad, Yazen Al-Hosni, Ronald Parsons, Jason Wellen, Su-Hsin Chang, Tarek Alhamad

**Affiliations:** ^1^ School of Medicine, Washington University in St. Louis, St. Louis, MO, United States; ^2^ Division of Nephrology, Department of Internal Medicine, Saint Louis University School of Medicine, St. Louis, MO, United States; ^3^ Division of Transplantation, Department of Surgery, Emory University School of Medicine, Atlanta, GA, United States

**Keywords:** allograft failure, kidney transplant, simultaneous pancreas-kidney transplantation, type 1 diabetes mellitus, allograft survival

In the published article, an author name was incorrectly written as “Krista Lentine.” The correct spelling is “Krista L. Lentine.”

The authors apologize for this error and state that this does not change the scientific conclusions of the article in any way. The original article has been updated.

