# Supplementary figures and images for: Survival After Simultaneous Pancreas‐Kidney Transplantation in Type 1 Diabetes: The Critical Role of Early Pancreas Allograft Function
Source: Transpl Int. 2022 Sep 12;35:10618. doi: 10.3389/ti.2022.10618 (PMC9510367; doi:10.3389/ti.2022.10618)

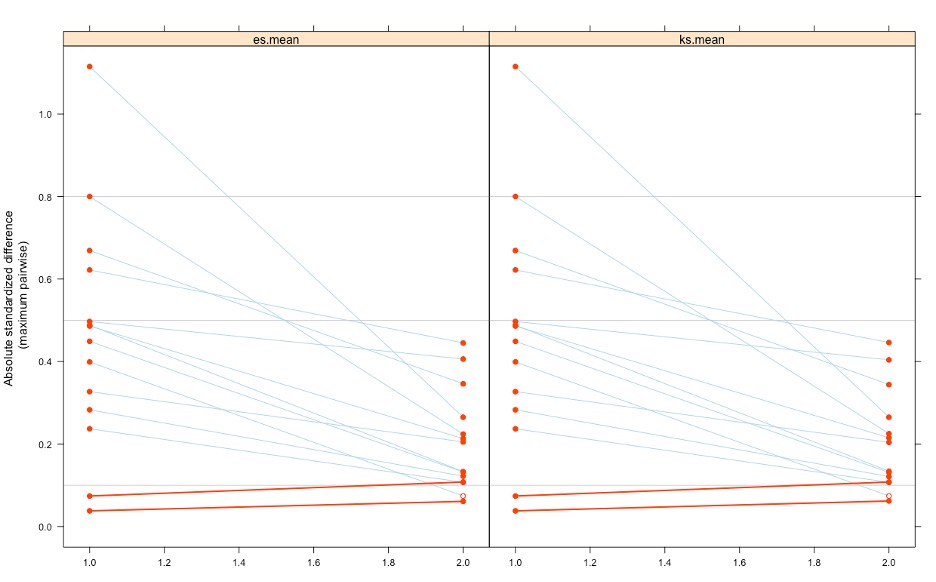

Supplement: Supplementary file 1 [file Image1.JPEG]
